# TAK1 Improves Cognitive Function *via* Suppressing RIPK1-Driven Neuronal Apoptosis and Necroptosis in Rats with Chronic Hypertension

**DOI:** 10.14336/AD.2023.0219

**Published:** 2023-10-01

**Authors:** Jing Yang, Pei Sun, Xiangming Xu, Xiaolu Liu, Linfang Lan, Ming Yi, Chi Xiao, Ruichen Ni, Yuhua Fan

**Affiliations:** Department of Neurology, The First Affiliated Hospital, Sun Yat-sen University; Guangdong Provincial Key Laboratory of Diagnosis and Treatment of Major Neurological Diseases, National Key Clinical Department and Key Discipline of Neurology, Guangzhou, China

**Keywords:** Hypertension, TAK1, neuron, apoptosis, necroptosis, RIPK1

## Abstract

Chronic hypertension is a major risk factor for cognitive impairment, which can promote neuroinflammation and neuronal loss in the central nervous system. Transforming growth factor β-activated kinase 1 (TAK1) is a key molecular component in determining cell fate and can be activated by inflammatory cytokines. This study aimed to investigate the role of TAK1 in mediating neuronal survival in the cerebral cortex and hippocampus under chronic hypertensive conditions. To that end, we used stroke-prone renovascular hypertension rats (RHRSP) as chronic hypertension models. Adeno-associated virus (AAV) designed to overexpress or knock down TAK1 expression were injected into the lateral ventricles of rats and the subsequent effects on cognitive function and neuronal survival under chronic hypertensive conditions were assessed. We found that, TAK1 knockdown in RHRSP markedly increased neuronal apoptosis and necroptosis and induced cognitive impairment, which could be reversed by Nec-1s, an inhibitor of receptor interacting protein kinase 1 (RIPK1). In contrast, overexpression of TAK1 in RHRSP significantly suppressed neuronal apoptosis and necroptosis and improved cognitive function. Further knockdown of TAK1 in sham-operated rats received similar phenotype with RHRSP. The results have been verified *in vitro*. In this study, we provide *in vivo* and *in vitro* evidence that TAK1 improves cognitive function by suppressing RIPK1-driven neuronal apoptosis and necroptosis in rats with chronic hypertension.

## INTRODUCTION

Hypertension is an epidemic health challenge, a confirmed main risk factor for stroke, cerebral small vessel disease (cSVD), and vascular cognitive impairment (VCI), and the leading global attributable risk for morbidity and mortality [1-4]. In 2000, more than a quarter of the global population was affected, and this figure will increase to 29%, by 2050, affecting nearly 1.56 billion people [5, 6]. Subsequent many population and prospective longitudinal studies have demonstrated a relationship between high blood pressure and cognitive impairment [7]. As such has been posited that exacerbated neuroinflammation and neuronal damage are linked to hypertension-induced cognitive impairment [7].

Recent studies have highlighted transforming growth factor β-activated kinase 1 (TAK1) as a central regulator of cell death and activated by a range of inflammatory cytokines [8-10]. Importantly, the indispensable role of TAK1 in cell survival and death was identified in previous studies using genetic and pharmacological inhibition [11, 12]. These studies revealed the physiological importance of TAK1 in maintaining cell viability and tissue homeostasis in multiple organs [12-14]. Down-regulation of TAK1 levels promote neuron death in the mouse brain following ischemic insult [14]. And in aging human brains, down-regulation of TAK1 levels have been shown to provide a potential mechanism that promote the onset of frontotemporal dementia/amyotrophic lateral sclerosis [15]. TAK1 has been showed to activate NF-κB, JNK/MAPK and p38/MAPK signaling pathways [8]. The most defining characteristic function of TAK1 is to mediate the activation of NF-κB signaling pathway, which mediates cell survival in some tissues [16], but recent studies have uncovered that deletion of TAK1 sensitizes cells to receptor interacting protein kinase 1 (RIPK1)-dependent apoptosis and necroptosis, independent of its role in the NF-κB pathway [17, 18].

RIPK1 has emerged as a key player in the regulation of cell death and inflammation [15, 17, 19], and has been implicated in the pathogenesis of ischemic injury, as well as neurodegenerative diseases such as Alzheimer’s disease [20], multiple sclerosis [21] and amyotrophic sclerosis [15]. In fact, several studies have demonstrated the physiological importance of RIPK1 in cell survival and death [14, 17]. Furthermore, there is growing evidences showing that inhibition of TAK1, TANK-binding kinase (TBK1), or NF-κB essential modulator (NEMO) promotes RIPK1 phosphorylation and sensitizes cells to RIPK1 dependent apoptosis or necroptosis independent of the NF-κB signaling pathway [15, 17, 22]. Autophosphorylation of RIPK1 on Ser166 is a critical incident regulating RIPK1-mediated cell fate [19]. Moreover, strong evidence implicates RIPK1 kinase activation induces cell death by activating caspase-8-caspase-3-dependent apoptosis and RIPK3-mixed lineage necrosis kinase-like (MLKL)-dependent necroptosis [23, 24].

Here, we propose the hypothesis that decreased TAK1 expression can induce neuronal apoptosis and necroptosis, which in turn leads to cognitive impairment in chronic hypertensive rats. We now report the fact that down-regulation of TAK1 in chronic hypertension rats can indeed induce the activation of neuronal apoptosis and necroptosis, in which a key regulator RIPK1 strictly regulates signal transduction.

## MATERIALS AND METHODS

### Animals

Male Sprague-Dawley (SD) rats were purchased from the Guangdong Medical Laboratory Animal Center (SCXK (Guangdong) 2022-0002). All animals were maintained under the environment of 12 h light/dark cycle and controlled temperature and humidity. All procedures were approved and implemented in accordance with the guidelines of the Institutional Animal Ethical Committee of Sun Yat-sen University and the Guide for the Care and Use of Laboratory Animals of the National Institute of Health in China.

### Animal models and experimental design

In total, 84 SD rats were randomly divided into sham-operated group (n=21) and stroke-prone renovascular hypertension rats (RHRSP) group (n=63). The RHRSP model was comprised of SD rats weighing 80-100 g exposed to a two-kidney two-clips method as previously described [25]. SD rats subjected to sham-operated group were treated similarly, except for clipping of the renal artery. Systolic blood pressure (SBP) was measured once a month after transverse renal artery contraction in preheated (37 °C, 15 min) conscious rats, using an indirect tail-cuff sphygmomanometer (BP-2010A, Softron, Japan). Twenty-four weeks after surgery, RHRSP rats with a stable SBP > 180 mmHg and without any stroke symptoms were selected. In total, 53 of the rats exposed to the two-kidney two-clips procedure met the experimental criteria.

**Figure 1.**
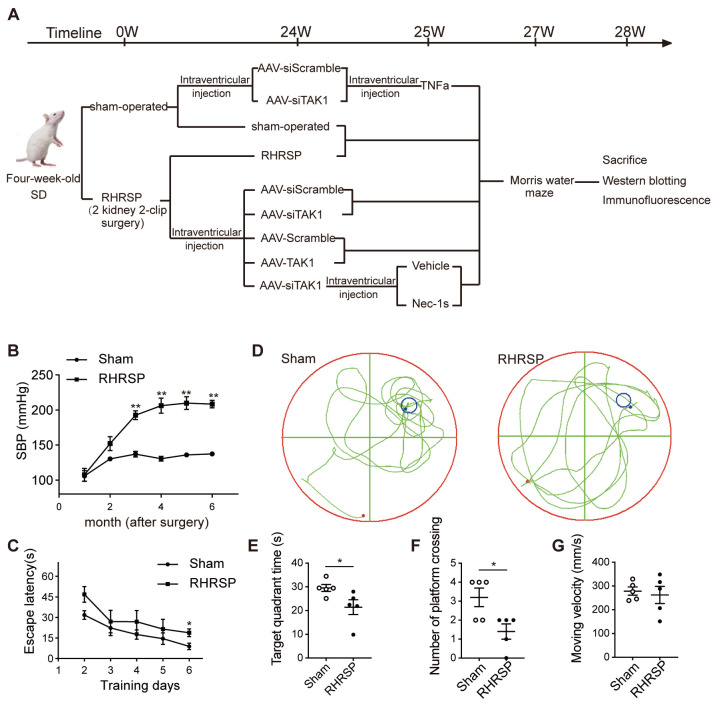
Increased systolic blood pressure induced by two-kidney two-clips surgery is associated with cognitive impairment in rats. (A) The diagram of treatment paradigms *in vivo* experiment. (B) Systolic blood pressure in the sham-operated and RHRSP rats. (C) The escape latency to reach the hidden platform on day 2-6 in the sham-operated and RHRSP rats. (D) The representative swimming traces of sham-operated and RHRSP rats. (E, F) The targe quadrant and the number of times crossing the original location of platform on the 7th day of probe trial in the sham-operated and RHRSP rats. (G) The moving velocity of sham-operated and RHRSP rats (n=5 per group). Data are expressed as mean ± standard error of mean (SEM). Non-parametric Mann-Whitney U test was used (*p < 0.05, **p < 0.01).

First, a group of RHRSP rats randomly received a stereotactically guided intraventricular injection of neuronal-specific adeno-associated virus vector (AAV) carrying either TAK1-ShRNA (referred to as AAV-siTAK1) (n=8) or Scramble-ShRNA (referred to as AAV-siScramble) (n=7) 24 weeks after renovascular hypertension surgery. Then, AAV-TAK1 (n=8) or AAV-Scramble (n=7) was stereotactically injected into the lateral ventricle of some randomly selected RHRSP rats. The remaining RHRSP rats were randomly selected for intraventricular administration of Nec-1s (a specific RIPK1 kinase inhibitor) (HY-14622A, MCE) or vehicle one week after the injection of AAV-siTAK1 (n=8 per group). Meanwhile, in order to further verify the role of TAK1, a group of sham-operated rats were randomly selected for intraventricular delivery of TNFα (HY-P7108, MCE) after a week of injection of AAV-siTAK1 or AAV-siScramble (n=7 per group). The diagram of treatment paradigms is shown in Figure 1A.

### AAV preparation and intraventricular injection

Three siRNA sequences targeting rat TAK1 (gene ID: 313121) and a negative control sequence were constructed by Hanbio (Shanghai, China). After being verified in primary rat neurons, the best-performing siRNA sequence was GCAAGAACTAGTTGCAGAA, and the negative control scramble siRNA sequence was TTCTCCGAAC GTGTCACGTAA. siRNAs were inserted into a pHB AAV-U6-MCS vector containing the U6 promoter upstream of the restriction sites (EcoRI (442) and BamHI (448)). Recombinant AAV was produced by transfecting 293T cells with Lipofiter™ (Hanbio Biotechnology, HB-TRCF-1000) according to standard protocols. Since AAV serotype 9 transduced the majority of neurons in central nervous system[26], it was used for transducing in this experiment. The virus titer, expressed as vector genomes (vg) per milliliter, was determined by measuring the genomic content of the AAV using the SYBRGreen method. The titer of AAV-siTAK1 was approximately 1.8*10^12 vg/ml.

The TAK1 and scrambled gene expression cassettes were inserted into a pHBAAV-CMV-MCS-3flag AAV vector containing a CMV promoter upstream of the restriction sites (EcoRI (740) and BamHI (2573)). The following treatment method was the same as described above, and the viral titer was determined after transfection into 293T cells, which was approximately 1.3*10^12 vg/ml. The AAV preparations were then stereotactically injected into bilateral lateral ventricles. Briefly, sham-operated and RHRSP rats were anesthetized with 1% pentobarbital (50 mg/kg) and AAV preparations were delivered into the bilateral lateral ventricles using a 15 μl Hamilton syringe coordinated by Bregma: anteroposterior -1 mm; mediolateral ±1.5 mm; dorsoventral -5.5 mm from the skull. A total of 15 μL of AAV was injected per site. The injection was performed at a rate of 2 μl/min and the needle was kept in place for 5 min before retraction. Rats were then removed from the stereotactic frame, sutured, and permitted to recover for 4 weeks in order to enable sufficient gene expression before being sacrificed or recovered for 1 week prior to Nec-1s, vehicle or TNFα treatment.

### Intraventricular administration of Nec-1s and TNFα

Nec-1s was dissolved in 10% DMSO (D2650, Sigma-Aldrich) and then transferred into 40% PEG300 solution, 5% Tween-80 and 45% saline, and finally diluted to a concentration of 25 mM. Some RHRSP rats that had been injected with AAV-siTAK1 for one week were randomly selected to be injected with Nec-1s into the right lateral ventricle again. A total of 200 μl clear solution was delivered to the lateral ventricle using a mini-osmotic pump (Alzet 2002, Alza Scientific Products) for 14 days at a rate of 0.5 μl per hour. TNFα was dissolved in 0.01 PBS and delivered to the right lateral ventricle at a dose of 200 μl (1 μl/ml) using mini-osmotic pumps.

### Cognitive function assessment

Twenty-seven weeks after operation, five rats were randomly selected from sham-operated and RHRSP groups to evaluate cognitive function using the Morris water maze test as previously described [27]. Three weeks after intraventricular administration, five rats were randomly selected from each of the groups (AAV-siTAK1, AAV-siScramble, AAV-TAK1, AAV-Scramble, sham+AAVsiScramble, sham+AAVsiTAK1, siTAK1+vehicle and siTAk1+Nec-1s) to evaluate cognitive function. On the first day, the rats were permitted to swim freely in the tank for three minutes for adaptive training. The spatial acquisition trials were then performed on days 2-6. The rats learned to navigate a direct path to the hidden platform using distal cues within no more than 60 s when starting from four different locations around the perimeter of the tank. On day 7, a probe trial was conducted to assess their reference memory at the end of learning, during which the platform was removed. The frequency of crossing the original position of the platform, the time spent in the target quadrant and the moving velocity were recorded within 60 s.

### Western blot analysis for in vivo experiment

The procedures were as described previously [28]. The cerebral cortex and hippocampus were collected from 0.9% saline-perfused brains removed during sacrifice, immediately frozen in liquid nitrogen, and stored at -80 °C until use. Brain tissues were homogenized in RIPA lysis buffer (R0010, Solarbio, China) containing phenylmethanesulfonyl fluoride (PMSF) and phosphatase inhibitor cocktail (4906837001, Roche, Switzerland) on ice, then centrifuged at 4 °C, 12000 g for 30 min, and the supernatant was collected for western blot analysis. The protein concentration was determined using a BCA protein assay kit (BCA-23225, Thermo Scientific, USA). Forty micrograms of protein extract from the cortex or hippocampus of each sample were separated by sodium dodecyl sulfate-polyacrylamide gel electrophoresis (SDS-PAGE) and then transferred to 0.22 μm polyvinylidene fluoride membranes (PVDF, ISEQ00010, Merck Millipore, USA). Then the membranes were blocked with non-fat milk (9999s, CST, USA) at room temperature for 60 min. Following primary antibodies incubated membranes were used: rabbit anti-cleaved caspase-3 (1:300, 9664S, CST, USA), rabbit anti-caspase-3 (1:1000, 9662S, CST, USA), rabbit anti-Phospho-MLKL (1:1000, 37333S, CST, USA), rabbit anti-MLKL (1:1000, ab184718, Abcam, USA), rabbit anti-GAPDH (1:2000, CST, USA), rabbit anti-TAK1 (1:5000, ab109526, Abcam, USA), rabbit anti-NEMO (1:5000, ab178872, Abcam, USA), rabbit anti-Phospho-RIP (Ser166) (1:1000, 53286S, CST, USA), rabbit anti-RIP (1:1000, 3493S, CST, USA), rabbit anti-Phospho-RIP3 (1:1000, 91702S, CST, USA), rabbit anti-RIP3 (1:1000, 10188S, CST, USA), rabbit anti-Cleaved caspase-8 (1:1000, 9496T, CST, USA), rabbit anti-caspase-8 (1:1000, ab32397, Abcam, USA), rabbit anti-Phospho-NF-κB p65 (1:1000, 3033S, CST, USA), rabbit anti-NF-κB p65 (1:1000, 8242S, CST, USA), rabbit anti-Phospho-JNK (1:1000, 4668S, CST, USA), rabbit anti-JNK (1:1000, 9252S, CST, USA), rabbit anti-Phospho-p38 MAPK (1:1000, 4511S, CST, USA), rabbit anti-p38 MAPK (1:1000, 8690S, CST, USA). After overnight incubation at 4 °C, the membranes were incubated with HRP-conjugated anti-rabbit antibody (1:3000, 5571s, CST, USA) at room temperature for 60 min. High-resolution digital images were captured by Amersham Imager 600 (USA) and relative densities were analyzed with ImageJ software (U.S. National Institutes of Health, Bethesda). The relative protein expression level was normalized to the intensity of the GAPDH or its corresponding total protein band.

### Immunofluorescent staining for in vivo experiment

Immunofluorescent staining was performed as described [29]. Anesthetized (1% pentobarbital) rats were sacrificed and perfused with normal saline followed by 4% paraformaldehyde, and 10 μm coronal brain-sections were prepared on a cryostat. The sections were blocked with 10% goat serum at room temperature for 60 min and incubated at 4 °C overnight with the following primary antibodies: mouse anti-NeuN (1:200, ab104224, Abcam, USA), rabbit anti-Phospho-RIP (1:100, 53286s, CST, USA), rabbit anti-Phospho-MLKL (1:200, 37333S, CST, USA), rabbit anti-cleaved caspase-3 (1:50, 9664S, CST, USA), and rabbit anti-TAK1 (1:100, ab109526, Abcam, USA), mouse anti-GFAP (1:400, MAB3402X, Millipore). After washing in 0.01M PBS (pH 7.4), the sections were incubated at room temperature for 60 min with the respective secondary antibodies: Alexa Fluor 647-conjugated goat anti-mouse IgG (1:1000, 4410S, CST, USA), and Alexa Fluor 488-conjugated goat anti-rabbit IgG (1:1000, 4413S, CST, USA). Isotype antibody controls and secondary antibody only controls employed to validate antibody specificity and distinguish genuine target staining from background. Fluorescence signals were then detected using fluorescence microscope (Olympus BX51, Tokyo). Images were acquired using Nikon NIS-Elements software. Every tenth coronal plane between -2.1 mm and -4.2 mm from bregma was selected in each group to perform immunofluorescent staining. Three planes of each rat and nine nonoverlapping 400xfields in the cortex, six fields of CA3 of hippocampus were selected for semi-quantitative analysis. For cortex, the number of immunostaining positive cells and colocalization of double-stained cells were counted with ImageJ software. But for hippocampus, it’s difficult to count the number of neurons, the percentages of area occupied by neurons and double-stained cells were calculated by ImageJ software.

### Cell culture and drug treatment

Hippocampal neuronal HT-22 cells were purchased from Procell (HT22) and maintained in DMEM (C1199 5500BT, GIBCO) containing 10% fetal bovine serum (0025, ScienCell) and 1% penicillin/streptomycin (0503, ScienCell) at 37 °C in humidity air with 5% CO2. To inhibit RIPK1 or TAK1, HT-22 cells were treated with 1ug/ml TNFα (HY-P7090A, MCE) in the absence or presence of 20 μM Nec-1s (HY-14622A, MCE) with/without 5 μM 5Z-7-Oxozeaenol (HY-12686, MCE). To inhibit NF-κB, 25 μM JSH-23 (HY-13982, MCE) was added to the cultures, meanwhile, TNFα was added to the culture medium. The final concentration was selected based on previously published literature [30-32]. After treatment, samples were prepared for cell viability analysis, protein extraction or fixed for immuno-fluorescent staining.

### Cell viability analysis

For cell viability analysis, 0.5-2 ×10^4^ cells were seeded per well of a 96 well plate before indicated treatment. Prior to experimental endpoints, cells were incubated with 110 μl of CCK8-mixture (1:10) (CK04, DOJINDO) for 1h and the absorbance at 460 nm was measured.

### Western blot analysis for in vitro experiment

The procedures were as described in tissue preparation in *vivo*. In brief, cell samples were lysed in RIPA lysis buffer containing PMSF and phosphatase inhibitor cocktail, and then cellular debris was removed by centrifugation at 4 °C, 12000 g for 30 min. Proteins (20 μg/sample) were separated on SDS-PAGE gels and transferred to 0.22 μm PVDF membranes. The densities of different protein bands were analyzed by ImageJ software. The experiments were repeated three times.

### Immunofluorescent staining for in vitro experiment

The procedures were the same as described *in vivo*. In brief, after treatment, cell samples were fixed with 4% paraformaldehyde for 15 min at room temperature and then incubated at 4 °C overnight with the primary antibodies. The next day, cells were incubated with corresponding secondary antibodies. Three independent experiments were conducted, and four random nonoverlapping 200xfields were selected for semi-quantitative analysis in each sample. The number of DAPI and colocalization of NeuN^+^Protein^+^-stained cells were counted with ImageJ software.

### Statistical analysis

All statistical analyses were performed using GraphPad Prism software (version 9.3.1). All data were expressed as mean ± standard error of mean (SEM). The difference between the two groups were analyzed by the non-parametric Mann-Whitney U test and multiple comparisons were analyzed by the non-parametric Kruskal-Wallis test. Further post-hoc analyses were conducted by comparing the mean ranks of preselected pairs of columns. Differences were considered statistically significant if *p* < 0.05 (*) or *p* < 0.01 (**). At least three independent biological repeats were presented at each data point.

**Figure 2.**
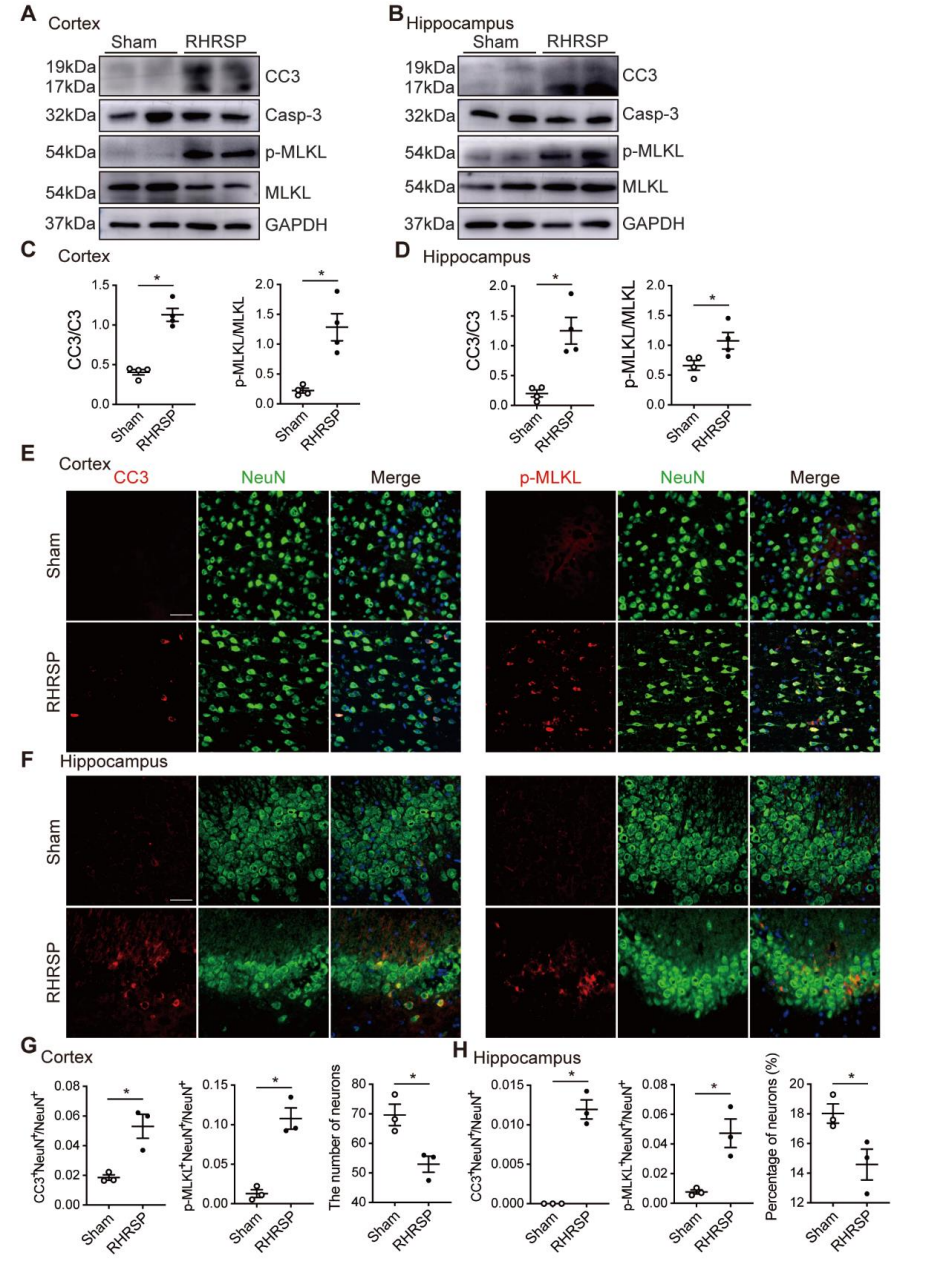
Neuronal apoptosis and necroptosis are activated in cerebral cortex and hippocampus of RHRSP. (A, B) The respective western blot images of cleaved caspase 3 (CC3) and p-MLKL in the cerebral cortex and hippocampus of sham-operated and RHRSP rats (n=4 per group). (C, D) Quantitative analysis of CC3 and p-MLKL levels (Protein levels were normalized to C3 and MLKL, respectively). (E, F) Co-staining of CC3 (red) or p-MLKL (red) with NeuN (green) in the cerebral cortex and CA3 regions of hippocampus of sham-operated and RHRSP groups. Scale bar: 50 μm. (G, H) Quantitative analysis of CC3^+^NeuN^+^ and p-MLKL^+^NeuN^+^ cells and the number or percentage of NeuN^+^ cells (n=3 per group). Data are expressed as mean ± SEM. Non-parametric Mann-Whitney U test was used (*p < 0.05).

## RESULTS

### Increased systolic blood pressure induced by two-kidney two-clips surgery is associated with cognitive impairment in rats

SBP was measured once a month after surgery. As shown in Figure 1B, the SBP of RHRSP rats gradually increased. The average SBP of RHRSP rats was significantly higher than that in the sham-operated group. To assess the impact of SBP on cognitive function, sham-operated and RHRSP rats were subjected to the Morris water maze test. Compared with sham-operated rats, RHRSP rats showed a longer escape latency to find the hidden platform (Fig. 1C). It was statistically significant on the 6th day. On the 7th day of probe trial, RHRSP rats spent less time in the targe quadrant and the number of times crossing the original location of platform was also reduced (Fig.1D-F), suggesting the impairment of cognition. But there was no difference in moving velocity between sham-operated and RHRSP rats (Fig. 1G).

**Figure 3.**
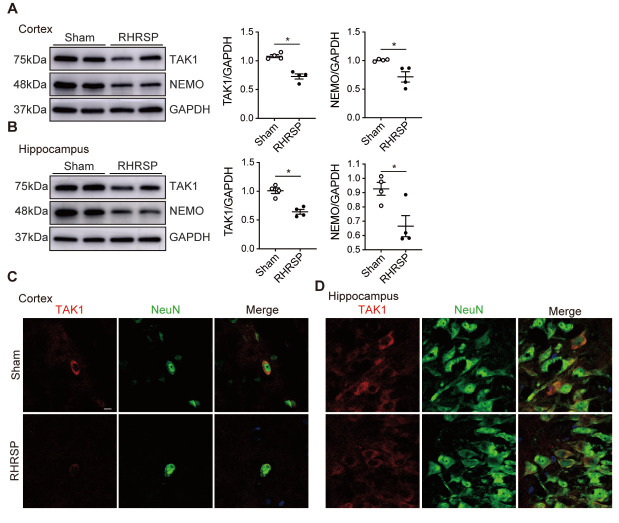
TAK1 expression is decreased in the cerebral cortex and hippocampus of RHRSP. (A, B) The respective western blot images of TAK1 and NEMO in the cerebral cortex and hippocampus of sham-operated and RHRSP groups (Protein levels were normalized to GAPDH) (n=4 per group). (C, D) Immunostaining of TAK1 (red) expression in NeuN^+^ neurons (green) in the cerebral cortex and hippocampus under chronic hypertensive condition. Scale bar: 10 μm. Data are expressed as mean ± SEM. Non-parametric Mann-Whitney U test was used (*p < 0.05).

### Neuronal apoptosis and necroptosis are activated in the cerebral cortex and hippocampus of RHRSP

To determine whether neuronal apoptosis and necroptosis were activated in the brains with chronic hypertension, we measured CC3 and p-MLKL in the cerebral cortex and hippocampus. Western blot analysis showed that the conversions of CC3 and p-MLKL were increased in the cerebral cortex and hippocampus of the RHRSP group relative to the sham-operated group (Fig. 2A-D). The immunofluorescence results revealed that there was a significant increase in double staining of CC3 with NeuN (neuronal nuclei, the marker for neurons) cells in the cerebral cortex and hippocampus of the RHRSP group compared to the sham-operated group (Fig. 2E-H), suggesting the occurrence of neuronal apoptosis following chronic hypertension. Meanwhile, there was an evident increase of p-MLKL^+^NeuN^+^ cells in the RHRSP group compared to that in the sham-operated group (Fig. 2E-H), indicating the development of neuronal necroptosis in the brains of chronic hypertensive rats. And the number of neurons in the cerebral cortex and hippocampus of RHRSP rats was lower than that of the sham-operated group (Fig. 2E-H). Thus, the chronic hypertension insult in rats triggers apoptosis and necroptosis in neurons, although it seems that more neurons undergo necroptosis.

### TAK1 expression is decreased in the cerebral cortex and hippocampus of RHRSP

Next, we explored the mechanisms promoting neuronal apoptosis and necroptosis in chronic hypertensive conditions. Since TAK1 is an important negative regulator of cell death[14, 29], we assessed whether deficiency of TAK1-mediated inhibition was involved in the process by detecting its expression level in the cerebral cortex and hippocampus of sham-operated and RHRSP rats. Western blot analysis showed that the expression of TAK1 in the cerebral cortex and hippocampus was decreased, as was the expression of NEMO (Fig. 3A, B), the downstream substrate of TAK1. We also assessed the expression of TAK1 in the neurons of cerebral cortex and hippocampus by immunostaining. Interestingly, we found that TAK1 expression was decreased in the brains of chronic hypertensive rats (Fig. 3C, D).

**Figure 4.**
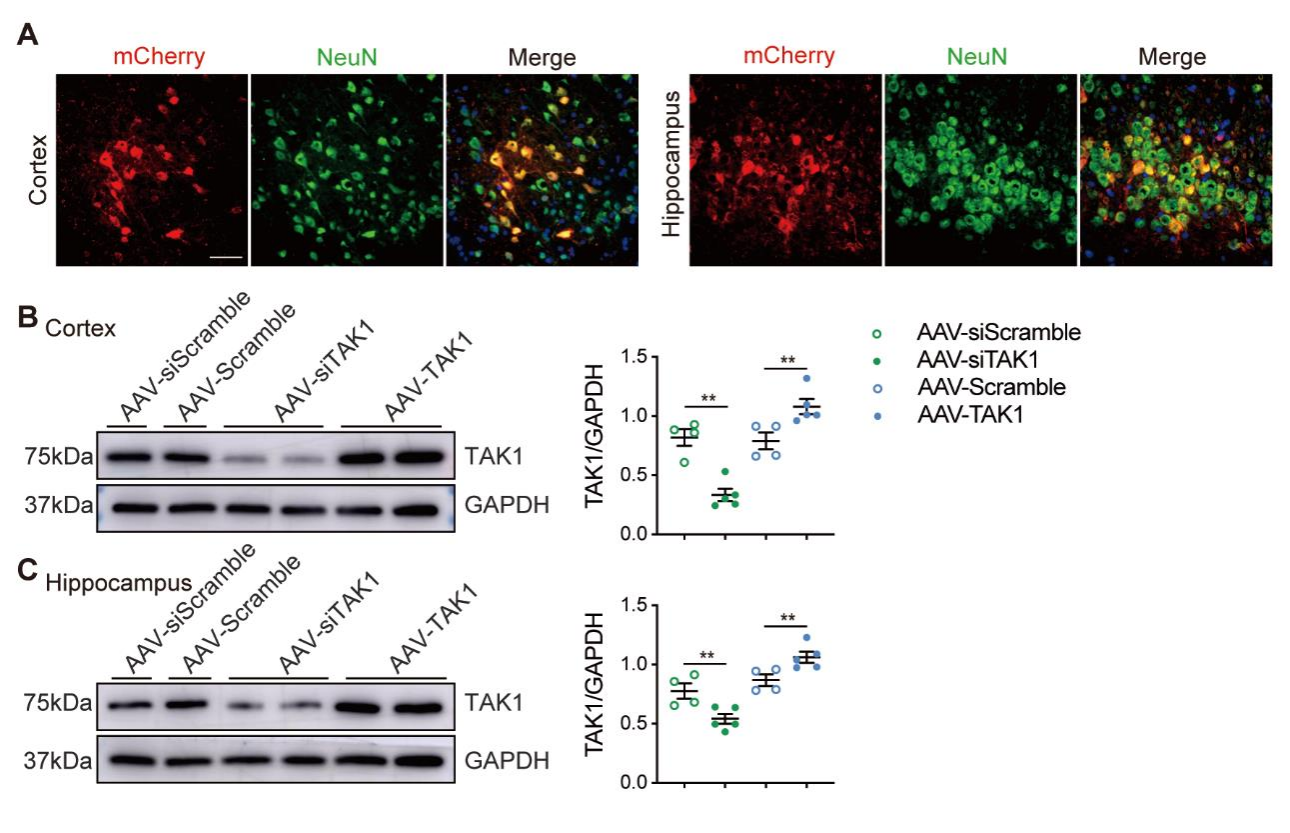
Knockdown or overexpression of TAK1 mediated by AAV in RHRSP. (A) mCherry-fluorescence (red) labelled AAV were detected in the cerebral cortex and hippocampus and coimmunostained with NeuN^+^ neurons (green). Scale bar: 50 μm. (B, C) The respective western blot images of the TAK1 in the cerebral cortex and hippocampus of AAV-siScramble (n=4), AAV-siTAK1 (n=5), AAV-Scramble (n=4) and AAV-TAK1 (n=5) groups (Protein levels were normalized to GAPDH). Data are expressed as mean ± SEM. Non-parametric Mann-Whitney U test used (**p < 0.01).

### TAK1 knockdown accelerates neuronal apoptosis and necroptosis in vivo

Based on the above data, we further hypothesized that deletion of TAK1 triggers neuronal death following chronic hypertension. To test this hypothesis, we injected the AAV encoding dominant negative TAK1 into the lateral ventricle of RHRSP rats to inhibit endogenous neuronal TAK1 activity. At the same time, a subset of RHRSP rats were injected with the control AAV expressing the scramble sequence. We found that four weeks after intracerebral ventricular AAV administration, mCherry-fluorescence-labeled AAV was predominantly distributed in the cerebral cortex and hippocampus. Almost all mCherry- fluorescence-labeled AAV colocalized with NeuN in the cerebral cortex and hippocampus (Fig. 4A). Although it has been reported that AAV9 can also transduced astrocytes[33, 34], we found almost no co-staining between mCherry and GFAP (Supplemetary Fig. 1A). The western blotting results revealed that TAK1 expression was markedly decreased in the cerebral cortex and hippocampus of rats injected with AAV-siTAK1 compared to those injected with AAV-siScramble (Fig. 4B, C).

**Figure 5.**
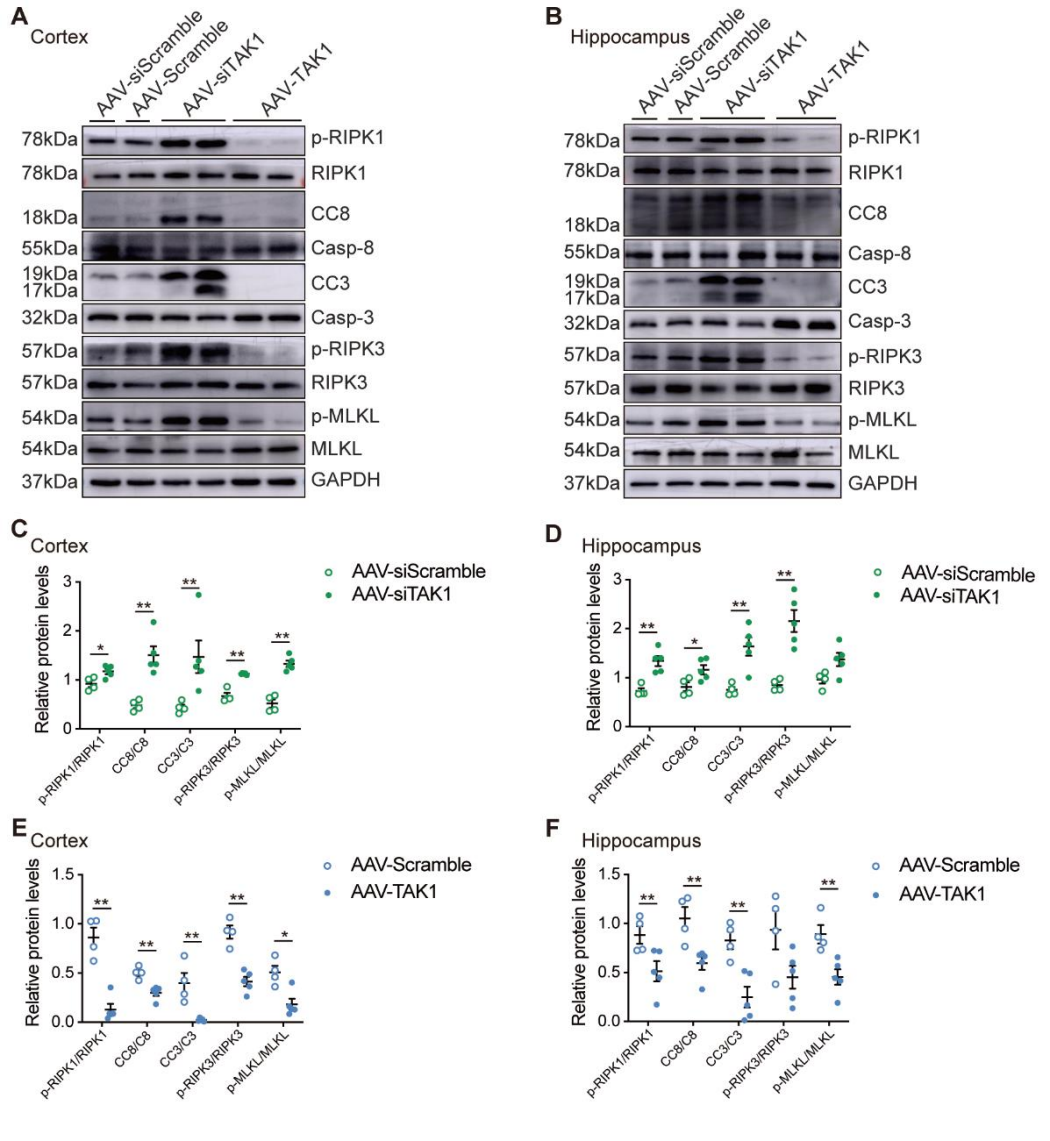
Apoptosis and necroptosis are increased after TAK1 knockdown, while decreased after TAK1 overexpression in RHRSP. (A, B) The respective western blot images of p-RIPK1, cleaved-caspase 8 (CC8), CC3, p-RIPK3 and p-MLKL in the cerebral cortex and hippocampus of AAV-siScramble (n=4), AAV-siTAK1 (n=5), AAV-Scramble (n=4), and AAV-TAK1 (n=5) groups. (C-F) Quantitative analysis of p-RIPK1, CC8, CC3, p-RIPK3 and p-MLKL levels (Protein levels were normalized to its corresponding total protein) in different groups. Data are expressed as mean ± SEM. Non-parametric Mann-Whitney U test was used (*p < 0.05, **p < 0.01).

**Figure 6.**
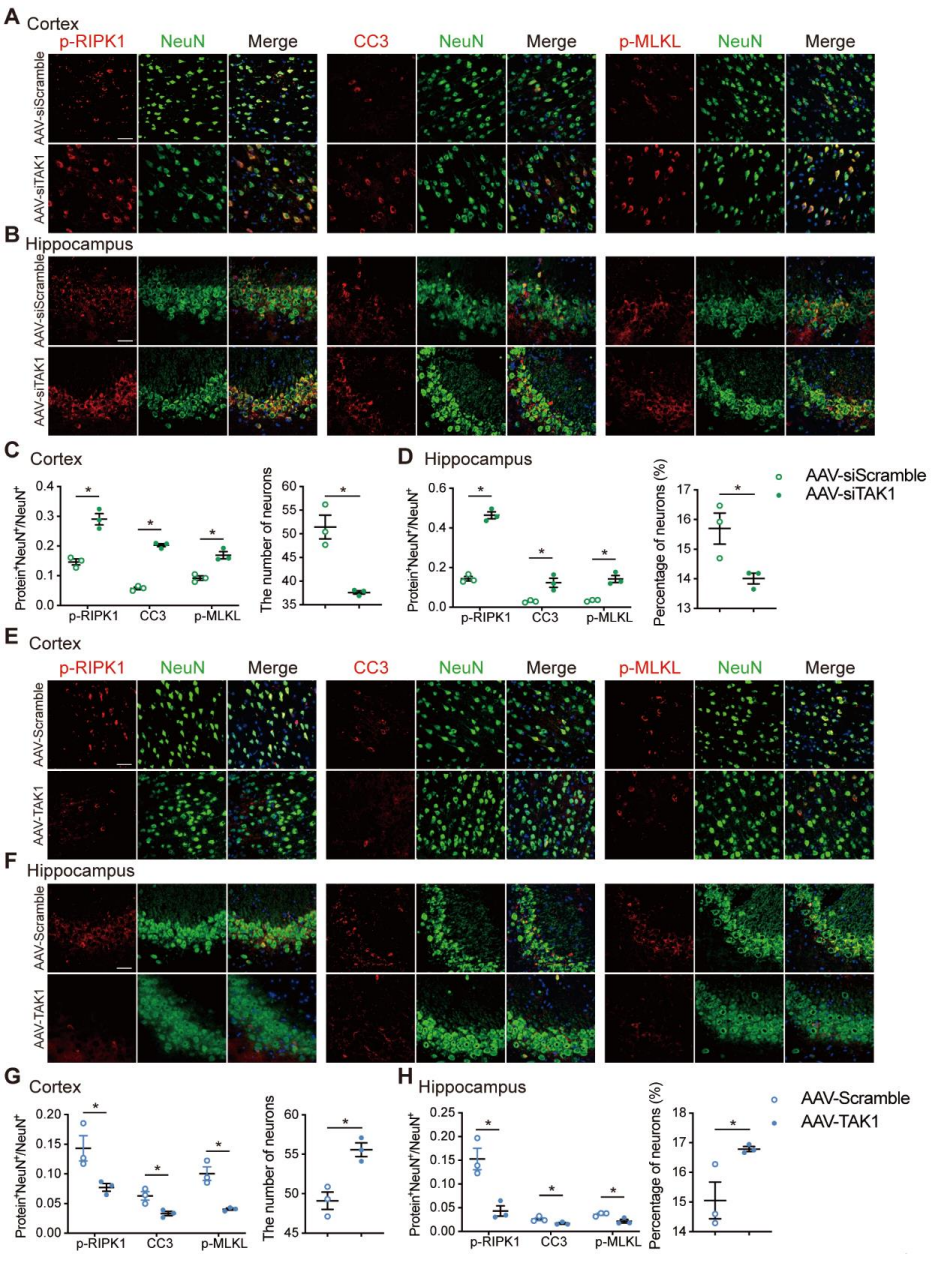
TAK1 knockdown or overexpression affects the survival and number of neurons in RHRSP. (A, B) Co-immunostaining of NeuN^+^ neuron (green) with p-RIPK1, CC3, p-MLKL (red) in the cerebral cortex and CA3 of hippocampus of AAV-siTAK1 and AAV-siScramble groups. Scale bar: 50 μm. (C, D) Quantitative analysis of p-RIPK1, CC3 and p-MLKL and the number or percentage of NeuN^+^ cells of AAV-siTAK1 and AAV-siScramble groups (n=3 per group). (E, F) Co-immunostaining of NeuN^+^ neuron (green) with p-RIPK1, CC3, p-MLKL (red) in the cerebral cortex and CA3 of hippocampus of AAV-TAK1 and AAV-Scramble groups. Scale bar: 50 μm. (G, H) Quantitative analysis of p-RIPK1, CC3 and p-MLKL and the number or percentage of NeuN^+^ cells of AAV-TAK1 and AAV-Scramble groups (n=3 per group). Data are expressed as mean ±SEM. Non-parametric Mann-Whitney U test was used (*p < 0.05).

Consistent with a previous study [14], our results also indicate that the deletion of TAK1 in neurons promotes cell death. Our western blotting results demonstrated that the level of phosphorylated RIPK1 at Ser166, a recognized marker for RIPK1 kinase activation [19], was markedly increased in AAV-siTAK1 group (Fig. 5A-D). Furthermore, compared to the AAV-siScramble group, the levels of cleaved caspase 8 (CC8), CC3, p-RIPK3, and p-MLKL were significantly increased in the cortex and hippocampus of AAV-siTAK1 group (Fig. 5A-D). Immunofluorescence staining of the cerebral cortex and hippocampus also revealed enhanced RIPK1 phosphorylation at Ser166 in neurons of AAV-siTAK1 group (Fig. 6A-D). Similarly, the number of CC3^+^NeuN^+^ and p-MLKL^+^NeuN^+^ cells in the cerebral cortex and hippocampus of the AAV-siTAK1 group was significantly higher than that in the AAV-siScramble-treated group (Fig. 6A-D). And the number of neurons in the cerebral cortex and hippocampus of the AAV-siTAK1 group was decreased (Fig. 6A-D). Similarly, the protein expression levels of NF-κB and MAPKs pathways were examined using western blot analysis. Our results showed that knockdown of TAK1 decreased the expressions of p-p65, p-JNK and p-p38 in the cortex and hippocampus (Supplemetary Fig. 1B-E). Taken together, these results indicate that TAK1 inhibition promotes both neuronal apoptosis and necroptosis in the cerebral cortex and hippocampus of chronic hypertensive rats.

### TAK1 overexpression inhibits neuronal apoptosis and necroptosis in vivo

To further confirm the role of TAK1 in the inhibition of neuronal apoptosis and necroptosis in chronic hypertensive rats, AAV-TAK1 was intraventricularly administered to RHRSP rats to induce TAK1 activation. Moreover, western blot analysis showed that the expressions of TAK1 in the cerebral cortex and hippocampus were significantly higher in the AAV-TAK1 group than in the AAV-Scramble group (Fig. 4B, C). Overexpression of TAK1 with AAV-TAK1 inhibited the cleavage of key regulators of the cell death pathways, including RIPK1, caspase 8, caspase 3, RIPK3, and MLKL (Fig. 5A, B, E, F), whereas the expressions of p-p65, p-JNK and p-p38 were increased in the cortex and hippocampus (Supplemetary Fig. 1B, C, F, G). Consistent with the western blot results, immunofluorescence staining also showed a significant decrease of neuronal apoptosis (CC3^+^NeuN^+^) and necroptosis (p-MLKL^+^NeuN^+^) in the cerebral cortex and hippocampus of rats treated with AAV-TAK1 (Fig. 6E-H). And there were more neurons in the AAV-TAK1 group than in the AAV-Scramble group (Fig. 6E-H).

### TAK1 knockdown induces neuronal death in sham-operated rats under the induction of TNFα

**Figure 7.**
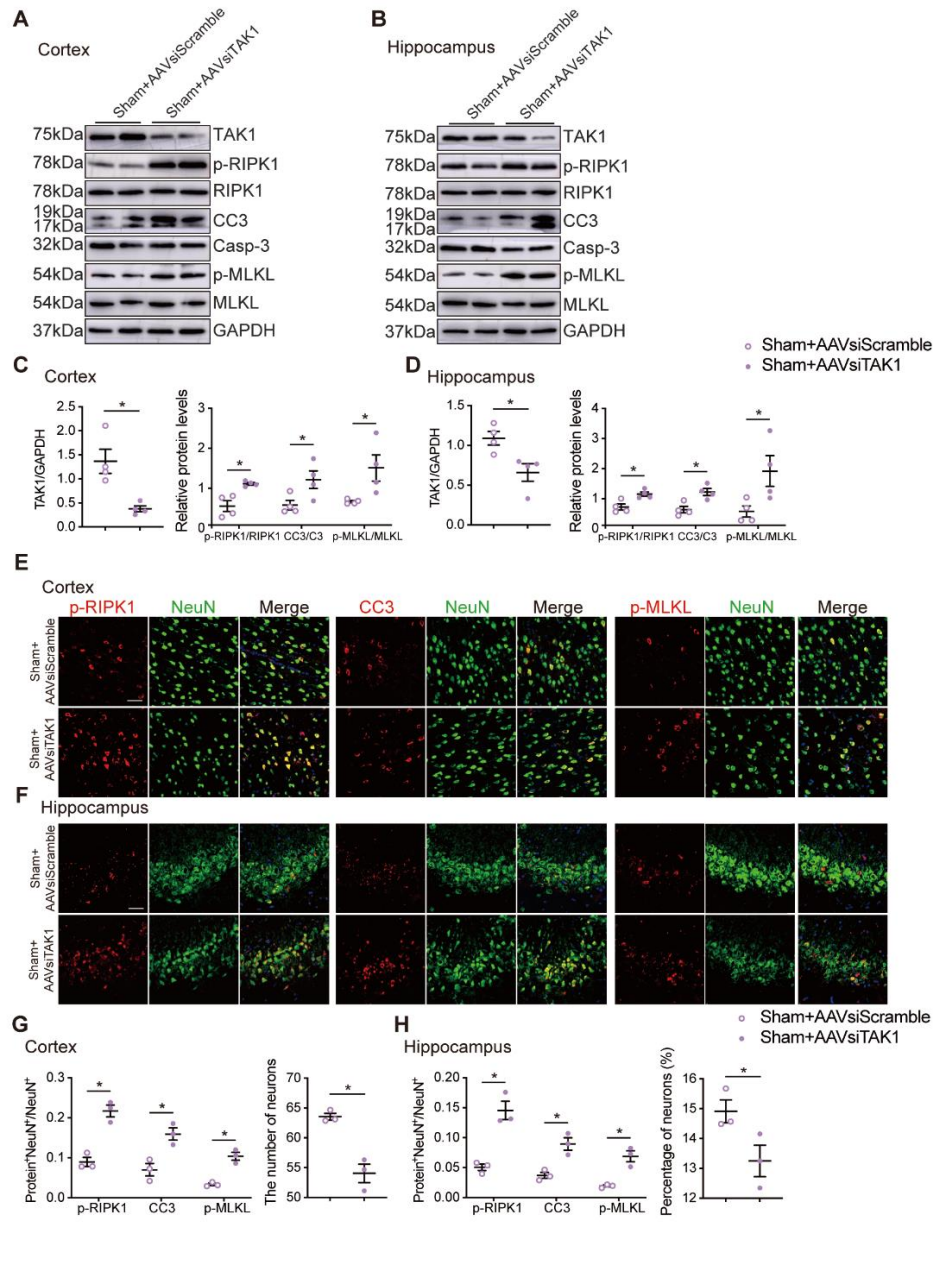
TAK1 knockdown induced neuronal death in sham-operated rats under the induction of TNFα. (A-D) Western blot analysis of cerebral cortex and hippocampus (sham+AAVsiScramble and sham+AAVsiTAK1 groups) (A, B) and the quantification of TAK1, p-RIPK1, CC3, and p-MLKL levels (TAK1 levels were normalized to GAPDH, p-RIPK1, CC3 and p-MLKL levels were normalized to its corresponding total protein) (n=4 per group) (C, D). (E, F) Co-immunostaining of NeuN^+^ neuron (green) with p-RIPK1, CC3, p-MLKL (red) in cerebral cortex and CA3 of hippocampus of sham+AAVsiScramble and sham+AAVsiTAK1 groups. Scale bar: 50 μm. (G, H) Quantitative analysis of p-RIPK1, CC3 and p-MLKL and the number or percentage of NeuN^+^ cells (n=3 per group). Data are expressed as mean ±SEM. Non-parametric Mann-Whitney U test was used (*p < 0.05).

To determine whether knock down TAK1 of sham-operated rats could receive similar phenotype with RHRSP, we knocked down TAK1 in sham-operated rats under the induction of TNFα. After administration of AAV-siTAK1, the expression of TAK1 was decreased (Fig. 7A-D) in the cortex and hippocampus of sham-operated rats. Similarly, the results showed that TAK1 knockdown markedly increased the apoptosis and necroptosis of neurons in the cortex and hippocampus, as confirmed by the increased expressions of p-RIPK1, CC3 and p-MLKL (Fig. 7A-D) and colocalization of NeuN^+^Protein^+^-stained cells (Fig. 7E-H). And the number of neurons in the cerebral cortex and hippocampus of the sham+AAVsiTAK1 group were decreased (Fig. 7E-H). We also detected the expressions of p-p65, p-JNK and p-p38 in the cortex and hippocampus using western blot analysis and found that their expressions were all decreased in the sham+AAVsiTAK1 group (Supplemetary Fig. 1H-K).

### TAK1 knockdown induced neuronal apoptosis and necroptosis is dependent on RIPK1 action

RIPK1 is a common factor in both the apoptosis and necroptosis pathways. To investigate whether the activation of neuronal apoptosis and necroptosis upon TAK1 deletion is dependent on the activation of RIPK1, we administered Nec-1s (a specific RIPK1 kinase inhibitor) to RHRSP rats that had received a cerebroventricular injection of AAV-siTAK1.

**Figure 8.**
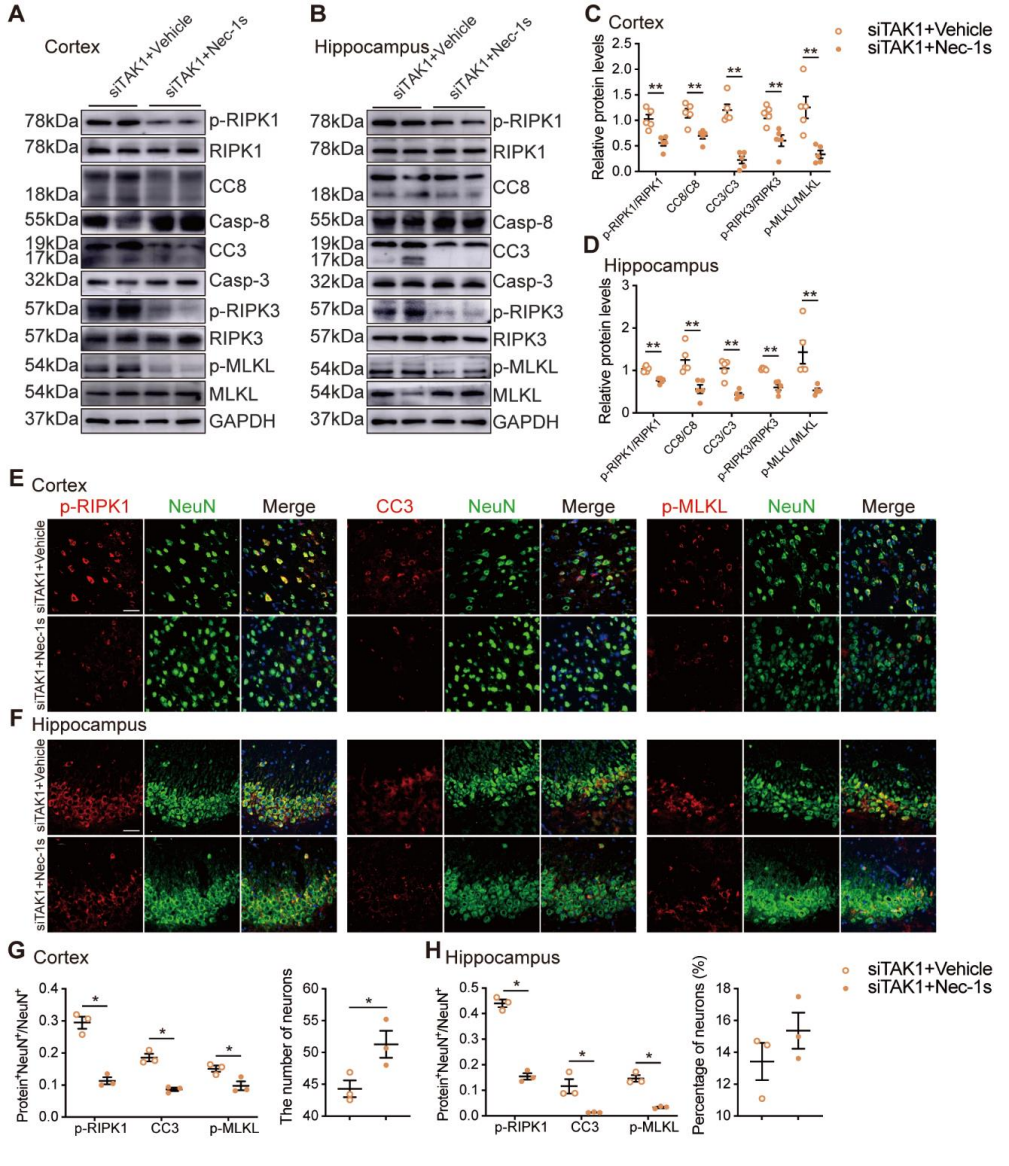
Nec-1s can reverse neuronal death and loss in the case of TAK1 knockdown in RHRSP. (A-D) Western blot analysis of cerebral cortex and hippocampus (siTAK1+Vehicle and siTAK1+Nec-1s groups) (A, B) and the quantification of p-RIPK1, CC8, CC3, p-RIPK3 and p-MLKL levels (Protein levels were normalized to its corresponding total protein) (n=5 per group) (C, D). (E, F) Co-immunostaining of NeuN^+^ neuron (green) with p-RIPK1, CC3, p-MLKL (red) in the cerebral cortex and CA3 of hippocampus of siTAK1+Vehicle and siTAK1+Nec-1s groups. Scale bar: 50 μm. (G, H) Quantitative analysis of p-RIPK1, CC3 and p-MLKL and the number or percentage of NeuN^+^ cells (n=3 per group). Data are expressed as mean ±SEM. Non-parametric Mann-Whitney U test was used (*p < 0.05, **p < 0.01).

According to the above results, significantly more neurons underwent apoptosis and necroptosis in the cerebral cortex and hippocampus of the AAV-siTAK1 group than in the AAV-siScramble group. Western blot analysis demonstrated that the levels of p-RIPK1, CC8, CC3, p-RIPK3, and p-MLKL were significantly decreased in the siTAK1+Nec1s group compared to the siTAK1+Vehicle group (Fig. 8A-D). Immuno-fluorescence staining also revealed that the number of CC3^+^NeuN^+^ and p-MLKL^+^NeuN^+^ cells in the cerebral cortex and hippocampus of the siTAK1+Nec1s-treated group were significantly decreased relative to the siTAK1+Vehicle-treated group (Fig. 8E-H). Both western blotting and immunofluorescence revealed that these effects could be reversed by Nec-1s, suggesting that RIPK1 is a key factor regulating neuronal apoptosis and necroptosis signaling under the conditions of TAK1 deficiency. At the same time, the number of neurons could also be reversed (Fig. 8E-H). Thus, inactivation of TAK1 promoted neuronal death via a RIPK1-dependent mechanism.

### Cognitive function

**Figure 9.**
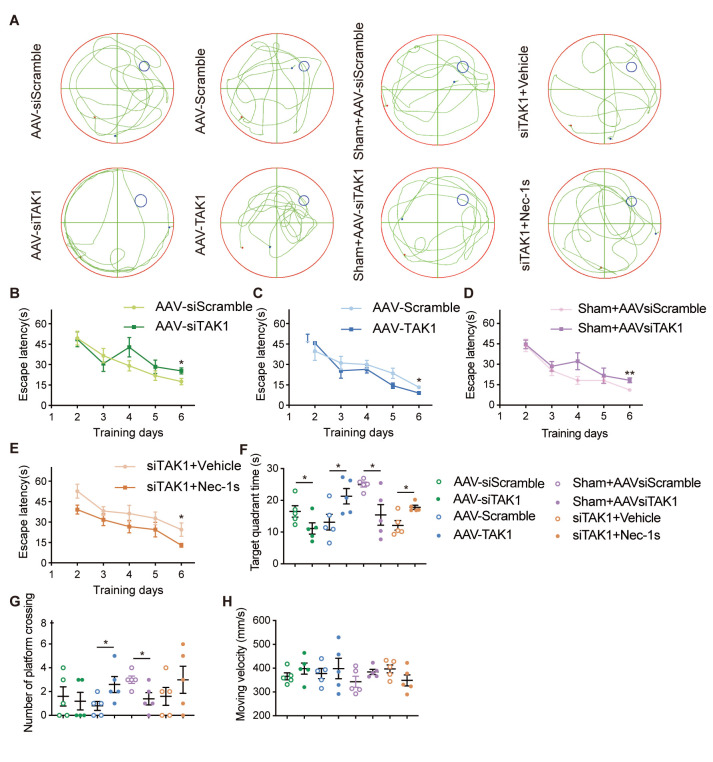
Cognitive function. (A) The representative swimming traces of the rats in the AAV-siScramble, AAV-siTAK1, AAV-Scramble, AAV-TAK1, sham+AAVsiScramble, sham+AAV-siTAK1, siTAK1+Vehicle and siTAK1+Nec-1s groups. (B-E) The escape latency to reach the platform on day 2-6. (F) Amount of time each group spent in the target area. (G) The number of times of crossing the platform in each group. (H) The moving velocity of each group. Data are expressed as mean ± SEM. n=5 per groups. Non-parametric Mann-Whitney U test was used (*p < 0.05, **p < 0.01).

The Morris water maze test results indicated that the rats in AAV-siTAK1 and sham+AAVsiTAK1 groups had longer escape latency to reach the hidden platform compared to their respective control groups on the 6th training day (Fig. 9B, D). In contrast, rats treated with AAV-TAK1 or siTAK1+Nec-1s had shorter escape latency than the AAV-Scramble or siTAK1+Vehicle groups after 6 days of training (Fig. 9C, E). On the 7th day of probe trial, rats of AAV-TAK1 and siTAK1+Nec-1s groups spent more time in the target quadrant compared with their respective controls, while the AAV-siTAK1 and sham+AAV-siTAK1 groups tended to shorten this time (Fig. 9A, F). As for the times of crossing the original platform location, AAV-siScramble, AAV-TAK1, sham+AAVsiScramble and siTAK1+Nec-1s groups all performed better than their respective control groups (Fig. 9A, G), although not all of them were statistically significant. And there was no difference in moving velocity between each control and experimental groups (Fig. 9H).

### TAK1 is essential for HT-22 cells survival under inflammatory conditions

**Figure 10.**
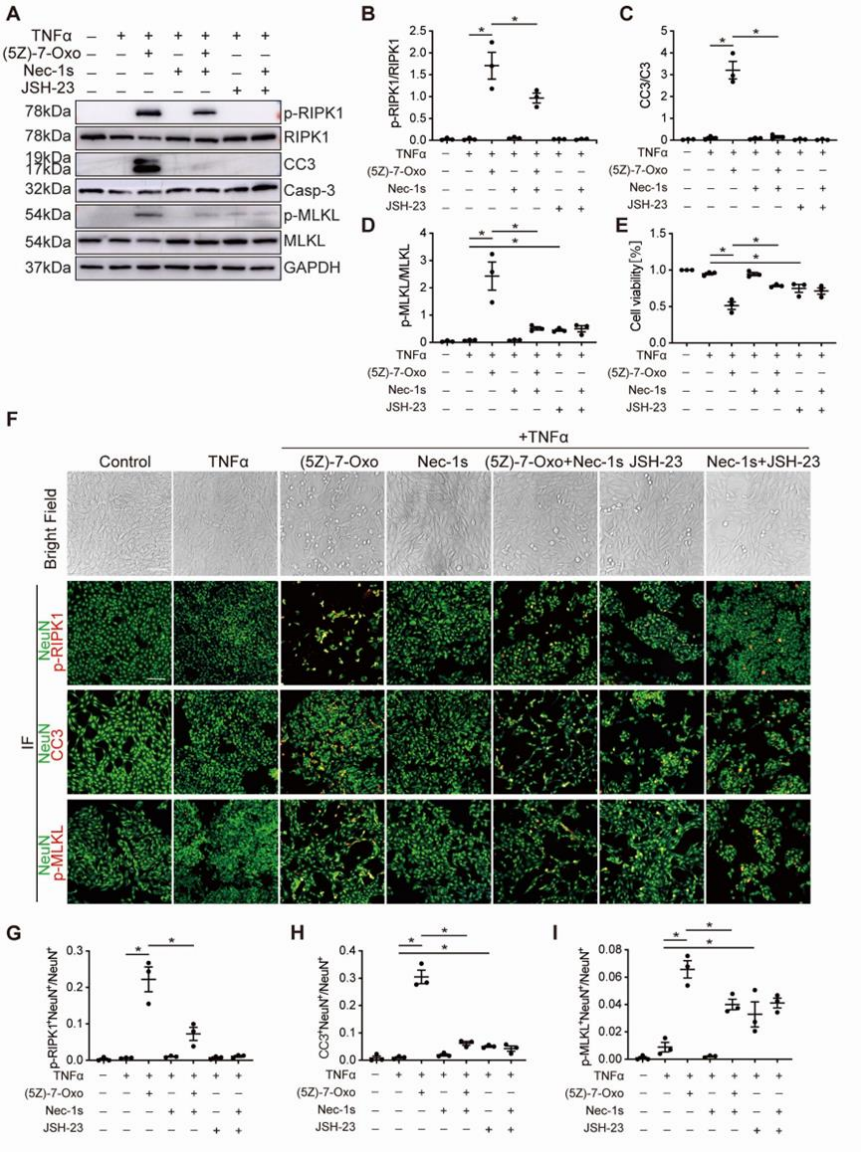
TAK1 is essential for HT-22 cells survival under inflammatory conditions. (A) Western blot shows the expression of p-RIPK1, CC3 and p-MLKL in HT-22 cells with or without indicated TNFα, 5Z-7-Oxozeaenol, Nec-1s and JSH-23 treatment. (B-D) Quantitative analysis of p-RIPK1, CC3 and p-MLKL levels (Protein levels were normalized to its corresponding total protein). (E) Quantitation of cell viability from HT-22 cell cultures with or without indicated treatment. (F) Images of bright field and immunostaining in HT-22 cells after indicated treatment. NeuN (green) as a neuronal marker. Scale bar: 100 μm. (G-I) Quantitation of p-RIPK1, CC3 and p-MLKL levels from NeuN-labeled cells in (F). n=3 independent cell culture experiments. Data are expressed as mean ± SEM. Non-parametric Kruskal-Wallis test was used (*p < 0.05).

To futher verify our conclusions, we have performed cell experiments corresponding to the *in vivo* models. We treated hippocampal neuronal HT-22 cells with several chemical inhibitors targeting the TAK1, RIPK1 and NF-κB together with TNFα, and found that TAK1 inhibitor 5Z-7-Oxozeaenol significantly increased the expressions of p-RIPK1, CC3 and p-MLKL in western blotting (Fig. 10A-D) and the number of p-RIPK^+^NeuN^+^, CC3^+^NeuN^+^ and p-MLKL^+^NeuN^+^ cells in immunostaining (Fig. 10F-I). And there were more apoptotic cells than necroptotic ones. Intriguingly, co-treatment with RIPK1 inhibitor Nec-1s could mostly reverse cell death induced by TAK1 inhibitor (Fig. 10A-I). Consistent with previous studies [35, 36], our research also found that NF-κB participated in the biological process of cell survival. We found that inhibiting NF-κB transcriptional activity by JSH-23, could also increase cell death, although its function was far less than that of TAK1 (Fig. 10A-I). But co-treatment with Nec-1s could not reverse cell death induced by JSH-23 (Fig. 10A-I). As expected, 5Z-7-Oxozeaenol suppressed NF-κB signaling, as demonstrated by reduction in p65 phosphorylation (Supplementary Fig. 1L, M). These results showed that the cell death caused by the inhibition of TAK1 is mainly due to the activation of RIPK1, rather than the inhibition of NF-κB pathway.

## DISCUSSION

The present study demonstrated that cognitive impairment, increased neuronal apoptosis and necroptosis in the cerebral cortex and hippocampus, and decreased TAK1 expression occurred under chronic hypertensive conditions. Notably, knockdown of TAK1, mediated by AAV-siTAK1, significantly increased RIPK1-caspase 8-mediated apoptosis and RIPK1-RIPK3-MLKL dependent necroptosis of neurons in the cerebral cortex and hippocampus, which was accompanied by the decrease of neurons and cognitive impairment. In contrast, activation of TAK1 with AAV-TAK1 markedly reduced neuronal apoptosis and necroptosis and prevented neuronal loss. Importantly, these effects were associated with an accelerated recovery of cognitive function. Furthermore, in the case of TAK1 deletion, treatment with Nec-1s reduced the expression of apoptosis and necroptosis related proteins and led to an increase in the number of neurons. At the same time, the cognitive deficits improved slightly. Taken together, the current study reveals a previously unrecognized biological function of TAK1 in regulating cognitive function and neuronal survival in chronic hypertensive rats, and the process was regulated by a RIPK1-mediated mechanism. The same conclusions were made by our*in vitro* experiment.

Many prospective longitudinal studies have established a relationship between elevated blood pressure and cognitive impairment [37-39]. Moreover, previous studies have shown that cognitive impairment related to the neuronal death in many diseases, such as Alzheimer’s disease [40, 41], VCI [42], and stroke [43] etc. In fact, at six months of age, unoperated spontaneously hypertensive rat (SHRSP) displayed a hypertension-induced neuronal death with reduced numbers [44], which was consistent with our findings. However, the molecular mechanisms underlying neuronal apoptosis and necroptosis in the cerebral cortex and hippocampus under chronic hypertension conditions have not yet been determined. Recent studies have shown that TAK1 signaling engages in multiple important biological processes, including inflammation, microglial activation, and cell survival [8, 29]. In the present study, we found that TAK1 expression was decreased in the cerebral cortex and hippocampus of RHRSP rats. These findings led us to hypothesize that the deletion of TAK1 might be involved in neuronal apoptosis and necroptosis in RHRSP rats. Therefore, knockdown or overexpression TAK1 by AAVs, which showed a corresponding increase or decrease of neuronal apoptosis and necroptosis in the cerebral cortex and hippocampus, respectively. Our study also confirmed the previously reported role of RIPK1 in driving cell death in TAK1 deficient mice [8, 17]. We provide the evidence that RIPK1 is a common signaling component of death receptor-mediated apoptosis and necroptosis through the regulation of two cell death-inducing complexes (RIPK1-caspase 8 and RIPK1-RIPK3-MLKL). Since neurons undergo apoptosis and necroptosis in the brains of chronic hypertensive rats simultaneously, inhibition of RIPK1 with Nec1-1s can block both apoptosis and necroptosis.

A previous study found that TAK1 expression in the mouse heart was downregulated under severe transverse aortic contraction [13]. Moreover, recent studies have reported a reduction of TAK1 expression following reperfusion after ischemia or aging human brains [14, 15]. They considered that the reduction of cerebral blood flow (CBF) in the brain is sufficient to promote the proteasome- and lysosome-mediated degradation of TAK1, thereby reducing the expression of TAK1. Intriguingly, previous studies have shown that CBF decreases in individuals with hypertension [45, 46]. In fact, focal CBF reduction can be observed in prefrontal lobe, cingulate gyrus, temporal lobe, and occipital cortex of patients with hypertension [47]. Therefore, it is reasonable to assume that hypertension can lead to hypoperfusion and initiate a chain reaction of TAK1 degradation in the brain. These results indicated that the loss of TAK1 is one of the primary pernicious mechanisms in the brain following chronic hypertension.

TAK1 is considered a key regulator of cell survival and death signaling, which is consistent with our observation that TAK1 deletion increases neuron death. Although the pro-survival biological function of TAK1 in different cell types has been well established, there is no consensus on whether overexpression of TAK1 can promote cell survival. For example, elevated expression of TAK1 in cardiomyocytes was observed to largely block cell death [13]. Additionally, overexpression of TAK1 in human melanoma cells may contribute to the prevention of cell death [31]. Another surprising finding showed that overexpression of TAK1 alone marginally enhanced fibroblast cell necroptosis [48]. It was also reported that deletion of Tab2 in cells lead to sustained TAK1 activation and exacerbated necroptosis [17, 48]. However, it is important to note that one of the above studies implied that inhibition of TAK1 by 5Z-7-Oxozeaenol could not prevent necroptosis in Tab2-depleted L929 cells [48]. In this study, we explored whether overexpression of TAK1 alone could enhance neuronal survival or not. Our result showed that TAK1 overexpression decreased neuronal apoptosis and necroptosis in the cerebral cortex and hippocampus, suggesting that sustained TAK1 signaling is essential for maintaining neuronal survival.

TAK1 has been showed to activate NF-κB, JNK/MAPK and p38/MAPK signaling pathways [8], at the same time, all of which play a role in neuronal death[49, 50]. Previous studies have showed that aberrant NF-κB, JNK/MAPK and p38/MAPK signaling could induce neuronal death and involve in the pathogenesis of many central nervous system diseases, such as Alzheimer’s disease [51-53] and stroke [54, 55]. In this study, we detected these proteins and found that their expressions changed when TAK1 was knocked down or overexpressed. Unfortunately, we did not explore whether these proteins would cause neuronal death in the context of chronic hypertension *in vivo*. NF-κB is a critical regulator of cell survival and development of inflammation. We briefly explored the role of NF-κB signaling in mediated neuronal survival *in vitro.* The results showed that the cell death caused by the inhibition of TAK1 is mainly due to the activation of RIPK1, rather than the inhibition of NF-κB pathway.

Although initially considered to be mutually exclusive cell states [17, 56], our study demonstrated that apoptosis and necroptosis can coexist under certain conditions. Consistent with our findings, cardiac-specific deletion of TAK1 has been showed to induce coexisting apoptosis and necroptosis in cardiac myocytes [13, 57]. Necroptosis can also be activated without the inhibition of apoptosis after the reduction of TAK1 under stroke conditions.

In conclusion, we provide*in vivo* and *in vitro* experimental evidence that TAK1 is essential for maintaining cognitive function and neuronal survival by suppressing neuronal apoptosis and necroptosis through a RIPK1-dependent mechanism in chronic hypertensive rats. Our results also suggest that TAK1 signaling, and its effectors may serve as novel targets for the treatment of cSVD and VCI caused by chronic hypertension.

## Supplementary Materials

The Supplementary data can be found online at: www.aginganddisease.org/EN/10.14336/AD.2023.0219.


