# Supplementary material for: TAK1 Improves Cognitive Function via Suppressing RIPK1-Driven Neuronal Apoptosis and Necroptosis in Rats with Chronic Hypertension
Source: Aging Dis. 2023 Oct 1;14(5):1799–817. doi: 10.14336/AD.2023.0219 (PMC10529759; doi:10.14336/AD.2023.0219)
Supplement: Supplementary file 1 [file AD-14-5-1799-s.pdf]

SUPPLEMENTARY DATA

TAK1 Improves Cognitive Function *via* Suppressing RIPK1-Driven Neuronal Apoptosis and Necroptosis in Rats with Chronic Hypertension

Jing Yang[#], Pei Sun[#], Xiangming Xu, Xiaolu Liu, Linfang Lan, Ming Yi, Chi Xiao, Ruichen Ni, Yuhua Fan[*]

© 2023. Yang J et al. Published online at http://www.aginganddisease.org/EN/10.14336/AD.2023.0219

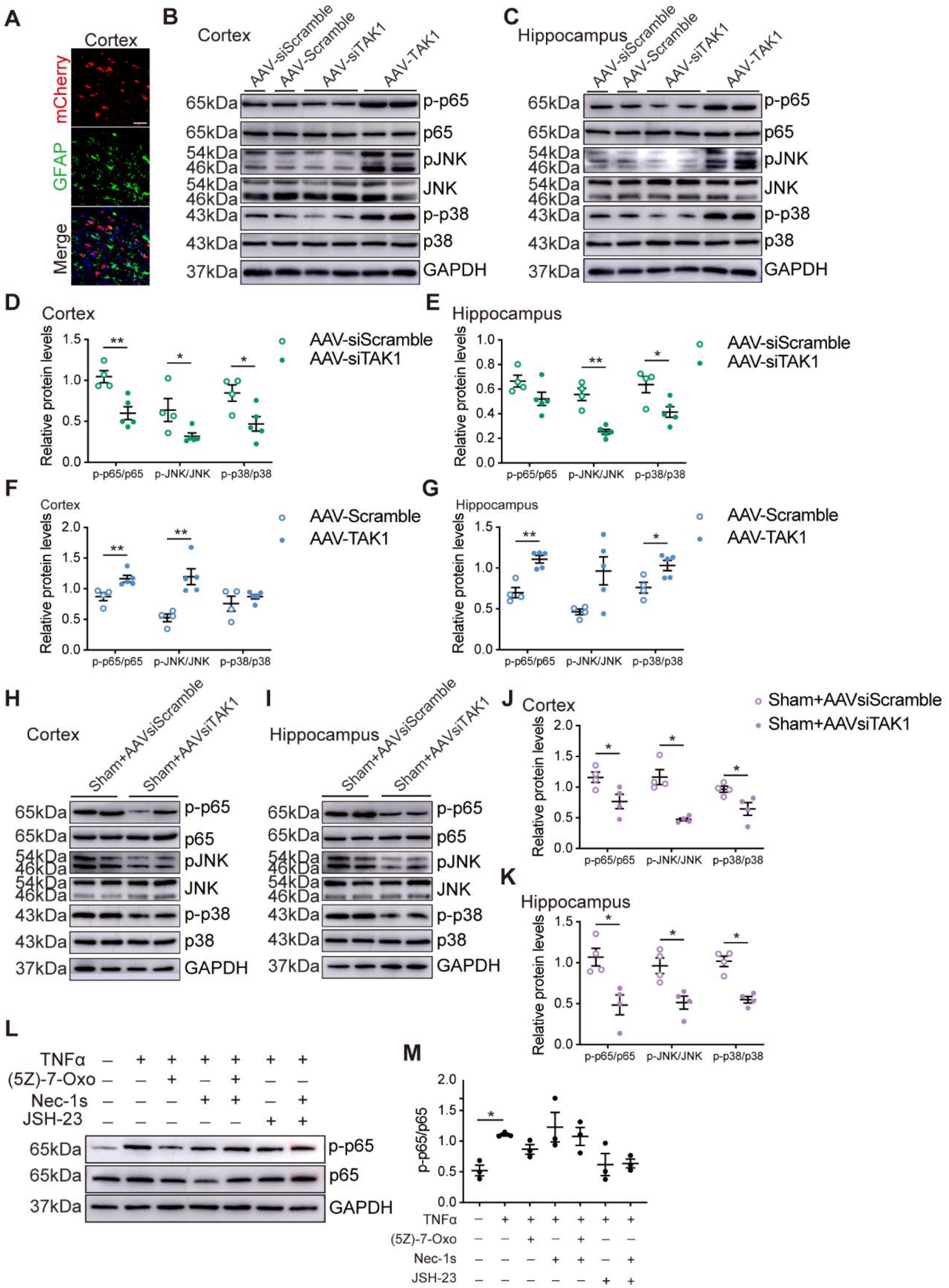

Supplementary Figure 1. The expression of p-p65, p-JNK and p-p38 in different groups. (A) Co-immunostaining of mCherry-fluorescence (red) and GFAP (green) in the cerebral cortex. Scale bar: 50 µm. (**B, C**) Western blot shows the expressions of p-p65, p-JNK, and p-p38 in the cerebral cortex and hippocampus of AAV-siScramble (n=4), AAV-siTAK1 (n=5), AAV-Scramble (n=4) and AAV-TAK1 (n=5) groups. **(D–G)** Quantitative analysis of p-p65, p-JNK, and p-p38 levels (Protein levels were normalized to its corresponding total protein). Data are expressed as mean ±SEM.

© 2023. Yang J et al. Published online at http://www.aginganddisease.org/EN/10.14336/AD.2023.0219

Non-parametric Mann-Whitney U test was used (*$p < 0.05$, **$p < 0.01$). (H, I) Western blot shows the expressions of p-p65, p-JNK, and p-p38 in the cerebral cortex and hippocampus of sham+AAVsiScramble and sham+AAV-siTAK1 groups (n=4 per group). **(J, K)** Quantitative analysis of p-p65, p-JNK, and p-p38 levels (Protein levels were normalized to its corresponding total protein). Data are expressed as mean ±SEM. Non-parametric Mann-Whitney U test was used (*$p < 0.05$, **$p < 0.01$). **(L)** Western blot shows the expression of p-p65 in HT-22 cells with or without indicated TNFα, 5Z-7-Oxozeaenol, Nec-1s and JSH-23 treatment. (M) Quantitative analysis of p-p65 levels (Protein levels were normalized to p65). n=3 independent cell culture experiments. Data are expressed as mean ± SEM. Non-parametric Kruskal-Wallis test was used (*$p < 0.05$).

© 2023. Yang J et al. Published online at http://www.aginganddisease.org/EN/10.14336/AD.2023.0219
